# Brain Responses to Acupuncture Are Probably Dependent on the Brain Functional Status

**DOI:** 10.1155/2013/175278

**Published:** 2013-05-21

**Authors:** Chuanfu Li, Jun Yang, Jinbo Sun, Chunsheng Xu, Yuanqiang Zhu, Qi Lu, Aihong Yuan, Yifang Zhu, Luoyi Li, Wei Zhang, Junping Liu, Jianjun Huang, Dongxiao Chen, Linying Wang, Wei Qin, Jie Tian

**Affiliations:** ^1^Laboratory of Digital Medical Imaging, The First Affiliated Hospital of Anhui University of TCM, Hefei, Anhui 230031, China; ^2^Life Sciences Research Center, School of Life Sciences and Technology, Xidian University, Xi'an, Shaanxi 710071, China; ^3^Key Laboratory of Complex Systems and Intelligence Science, Institute of Automation, Chinese Academy of Sciences, P.O. Box 2728, Beijing 100190, China

## Abstract

In recent years, neuroimaging studies of acupuncture have explored extensive aspects of brain responses to acupuncture in finding its underlying mechanisms. Most of these studies have been performed on healthy adults. Only a few studies have been performed on patients with diseases. Brain responses to acupuncture in patients with the same disease at different pathological stages have not been explored, although it may be more important and helpful in uncovering its underlying mechanisms. In the present study, we used fMRI to compare brain responses to acupuncture in patients with Bell's palsy at different pathological stages with normal controls and found that the brain response to acupuncture varied at different pathological stages of Bell's palsy. The brain response to acupuncture decreased in the early stages, increased in the later stages, and nearly returned to normal in the recovered group. All of the changes in the brain response to acupuncture could be explained as resulting from the changes in the brain functional status. Therefore, we proposed that the brain response to acupuncture is dependent on the brain functional status, while further investigation is needed to provide more evidence in support of this proposition.

## 1. Introduction

Acupuncture is an ancient East Asian healing modality that has been in use for more than 2000 years [1, 2]. In recent years, it has gained great popularity as an alternative and complementary therapeutic intervention in modern society [3]. However, the physiological mechanisms underlying its effects are still unclear, and various controversies remain [4–7].

Recent evaluation of this treatment modality has lent credence to the hypothesis that the brain and nervous system play a leading role in processing acupuncture stimuli. To find its underlying mechanism, extensive aspects of brain responses to acupuncture have been explored with neuroimaging techniques. Most of the acupuncture neuroimaging studies have been performed in healthy adults [8–43]. Only a few studies have been performed in patients with different diseases, and the related acupuncture mechanisms were explored [44–49]. However, brain responses to acupuncture at different pathological stages of the same kind of disease, which may be more important in uncovering its underlying mechanisms, have not yet been explored. Therefore, a study on the brain response to acupuncture at the different stages of Bell's palsy will be helpful in explaining the mechanisms of acupuncture.

To probe into this question, a certain type of disease should be selected for this study, which ought to be a common disease, and acupuncture is the most commonly used treatment choice. In China, acupuncture is frequently used to treat diseases and disorders of the nervous system such as stroke, dementia, Parkinson's disease, epilepsy, carpal tunnel syndrome, headache, and Bell's palsy [2]. Although some studies concluded that there is inadequate evidence to support the effectiveness of acupuncture for Bell's palsy [50, 51], a number of other studies have provided evidence that acupuncture is beneficial [2, 52, 53] or, as reported [54], that the efficacies of acupuncture, steroids, and natural course of recovery in Bell's palsy were the same with respect to the degree of recovery and speed of recovery. More importantly, Bell's palsy is a transient peripheral motor disease from which most of the patients would recover in several months and in which the brain anatomical structure was not significantly altered [55, 56]. The only changes in the brain that had been reported were the changes in the brain functional status or connectivity in the patients with Bell's palsy. Therefore, Bell's palsy would be an ideal disease for observing the changes in the brain response to acupuncture at different pathological stages. In this study, we explored the brain responses to acupuncture at different pathological stages of Bell's palsy and compared them with those in healthy controls in order to find whether or not the brain responded to acupuncture differently at different pathological stages, and to explore the possible underlying mechanisms of the brain response to acupuncture.

## 2. Materials and Methods

### 2.1. Subject Recruitment and Retention

All subjects, including healthy volunteers and patients with Bell's palsy, signed informed consents in accordance with the Human Research Committee of the First Affiliated Hospital of Anhui University of Traditional Chinese Medicine before they took part in the experiment. The fMRI data processed in this paper was only a part of the data collected in the Project for the National Key Basic Research and Development Program of China (Grant no. 2010CB530500). In this project, a total of 63 cases with Bell's palsy and 39 cases of healthy volunteers were recruited. All patients were right handed, with no central nervous system diseases, no mental diseases, and no other serious diseases. The healthy volunteers were college students or the workers in the hospital, who were also right handed with no history of mental or neurological disease and with no history of psychiatric disorders or drug use.

In accordance with the project's scheme, the patients were randomly divided into two study groups, 28 cases in the ipsilateral acupuncture group and 35 cases in the contralateral acupuncture group for another study objective (laterality of acupuncture). In the ipsilateral acupuncture group, the acupuncture was performed on the same side as that of facial palsy when acupuncture fMRI data was being acquired, while the acupuncture was performed on the opposite side as that of facial palsy in the contralateral acupuncture group. In this paper, only parts of the fMRI data of contralateral acupuncture group were analyzed. According to the scheme of the project, the healthy volunteers were requested to take part in the experiment only once, while the patients were asked to take part in the experiment three times, which was performed before the acupuncture treatment, during the treatment, and after recovery from Bell's palsy, respectively. 

The fMRI data acquisition before treatment was performed before any acupuncture treatment was done on the patient. After that, the patients received acupuncture treatment three times a week. A semi-individualized approach was used in the acupuncture treatment, and every subject was treated by manual needling at acupoints chosen by the acupuncture specialist according to the individual symptoms. The fMRI during the treatment was performed in about 2–4 weeks after acupuncture treatment had begun. However, if the recovery process was very slow, the fMRI during the treatment would be postponed to some extent. After the patients had fully recovered from the disease (the HB scale returned to grade I), the last fMRI data acquisition would be done. However, because the compliance of the patients was not the same, the actual times of the experiment were different. Among the 35 cases of patients, 4 cases of patients took part only once in the experiment, 2 times in 16 cases, 3 times in 13 cases, and 4 times in 2 cases. A total of 83 times of the fMRI examination were performed in patients. Except for 5 times of acquisition failure due to obvious movement of the participants, a total of 78 times of acupuncture fMRI data acquisition were successfully completed in the patients. An additional 39 times of data acquisition were successfully collected in normal controls.

### 2.2. Relevant Information Acquisition

The House-Brackmann facial nerve grading system (HBS), which is the most widely applied scoring system (1 = normal facial movement; 6 = no movement) [57], was used for clinical assessment of facial muscle function. Before the experiment, general information such as gender, age, duration from onset, HBS scale, and current symptoms was recorded into the case report form. After the experiment, the participants were questioned in detail about the severity of each kind of acupuncture sensation they felt including soreness, numbness, fullness, aching, spreading, and heaviness, and each kind of acupuncture sensation was scored based on 4 grades (0 = no sensation felt, 1 = mild, 2 = moderate, and 3 = severe). 

### 2.3. Data Acquisition of Acupuncture fMRI and Stimulation Protocol

Before the experiment, participants were asked to change clothes, rest, and then enter into the scanning room after their whole body was relaxed. The subjects were told to lie down and close their eyes as much as possible. Their ears were packed with cotton balls. The lights in the scanning room were turned off to reduce visual stimulation. During the entire scanning process, the subjects were told to avoid psychological activity as much as possible. 

All fMRI experiments were completed in the MRI room of the Medical Imaging Center, the First Affiliated Hospital of Anhui University of TCM. The Siemens Symphony 1.5 T MRI whole body scanner and standard head coil were used. A total of eight sequences were scanned, which were as follows. 

(1) Pilot images (2) T2-weighted images which to find whether or not there was any obvious disease of the brain. (3) T1-weighted 2D anatomical images which obtained the axial position parallel to the AC-PC line with a total of 36 slices that covered the whole brain. T1-weighted spin-echo sequence was used, with TR/TE of 500/12 ms, FOV of 230 mm × 230 mm, slice thickness/interval of 3.0 mm/0.75 mm, and resolution of 192 × 144. (4) Resting state fMRI before acupuncture. (5) Resting state fMRI during acupuncture; (6) Resting state fMRI after acupuncture. (7) Task-state acupuncture fMRI: EPI-BOLD sequence was used. The scanning direction and the number of slices were the same as those of the 2D anatomical images, with TR/TE/FA of 4000 ms/50 ms/90°, FOV of 192 mm × 192 mm, and resolution of 64 × 64. and (8) T1-weighted 3D anatomical images: the sagittal position was taken, and a total of 176 slices were scanned which covered the whole brain. The spoiled gradient echo sequence was used, with TR/TE/FA of 2100 mm/3.93 mm/13°, FOV of 250 mm × 250 mm, slice thickness/spacing of 1.0 mm/0.5 mm and resolution of 256 × 256. It took about 60 minutes to complete all of the data acquisition.

**Figure 1:**
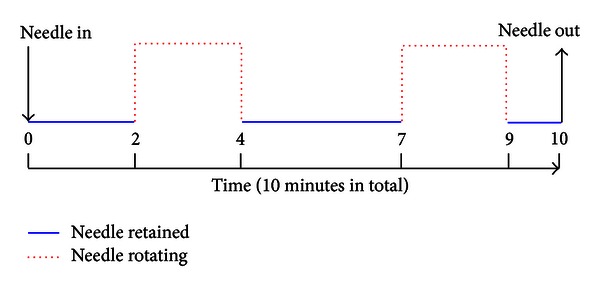
Diagram for the acupuncture stimulation protocol. The needle was inserted into the skin before fMRI data acquisition. After remaining at rest for 2 min, the needle was rotated bidirectionally with an even motion at the rate of 1 Hz for 2 min. After another rest period of 3 min, needle manipulation was repeated in a similar manner for another 2 min. After that, scanning continued for 1 min. The needle was pulled out at the end of the 10 min.

Only the data of task-state acupuncture fMRI was analyzed in this paper. A modified block design was used in the task-state acupuncture fMRI experiment which was the same as in Hui's study [11]. Disposable sterile stainless steel needles were used for the acupuncture at Hegu (LI4) on the contralateral side of the facial palsy. Just before fMRI data acquisition, the needle was inserted into the skin and manipulated until a *de-qi* sensation was obtained, and then the needle was retained and fMRI data acquisition began. Stimulation consisted of rotating the needle bidirectionally with an even motion to an amplitude of approximately 180° at the rate of one cycle per second, and the needle insertion depth was about 1.0 cm. After remaining at rest for 2 min, the needle was rotated bidirectionally with an even motion at the rate of 1 Hz for 2 min. After another rest period of 3 min, needle manipulation was repeated in a similar manner for another 2 min. After that, scanning continued for 1 min (see Figure 1). The needle was removed at the end of the 10 min experimental run. All operations of acupuncture were performed by a professional acupuncturist. 

### 2.4. Data Grouping and Exclusion

Subjects were classified into four groups including healthy volunteers (hereafter referred to as the normal group), patients at early stages (early group), patients at later stages (later group), and patients recovered from Bell's palsy (recovered group). The criteria for grouping patients with Bell's palsy were as follows. Recovered group: the HBS scale was equal to grade I no matter how long the duration from the onset of Bell's palsy was. Later group: the duration was more than two weeks, and the HBS scale was larger than grade I. Early group: the duration was less than two weeks, and the HBS scale was larger than grade I. Normal group: age- and gender-matched healthy volunteers. 

According to the grouping criteria, there were 23 times of fMRI acquisition data which fell into the recovered group, 27 in the later group, 28 in the early group, and 39 in the normal group. To make the sample size of the normal group similar to the patient group and the age of the normal group similar to the other three groups, 11 times of data acquisition from younger normal controls were dropped. Furthermore, in order to avoid the problem that the same subject was included in the same group more than once, only the first time of the acquisition data was kept if the same patient was included in the same group two or more times. As a result, 8 times of the acquisition data in the later group were discarded. The reasons for that all of the repeated data were from the later group are described as follows. Some patients took part in the experiment 14 days after the onset, and some patients had not yet fully recovered when the third or even the fourth times of data acquisition were performed. After considering all of these exclusions, the final sample size was 23 in the recovered group, 19 in the later group, 28 in the early group and 28 in the normal group.

### 2.5. Individual Analysis of fMRI Data

Data analysis was performed using AFNI software (http://afni.nimh.nih.gov/afni/download/afni/) in the Laboratory of Digital Medical Imaging, the First Affiliated Hospital of Anhui University of TCM. Initially, the first 4 time points were discarded in order to avoid the instability of the initial MRI signal. The remaining images were then realigned to the first volume. Thereafter, the images were normalized to the standard Talairach atlas and then smoothed spatially using a 6 mm full width at half maximum (FWHM) Gaussian kernel to decrease spatial noise. Global intensity normalization was not applied according to Sun's report [58]. The time series from each voxel was detrended using the method of linear least squares (3dDetrend, AFNI) to remove low-frequency noise and signal drift. For each subject, the preprocessed fMRI data were then submitted for analyses using the general linear model (3dDeconvolve, AFNI), and the *coef* value in the individual analysis results was extracted as the contrast image for further analysis. 

### 2.6. Group Analysis and Intergroup Analysis

In order to avoid the possible influence of head movement on the results of the data analysis, the data where head movement was more than 2 mm or 2° were excluded. As a result, 1 case was excluded in the normal group, 5 cases in the early group, and 1 case in the recovered group. The final sample size was 27, 23, 19, and 22 in the normal, early, later, and recovered groups, respectively. Before the group analysis, data collected from patients with left-sided facial palsy were mirror reversed across the midsagittal plane. Therefore, all group analysis results were equivalent to the results of right-sided facial palsy with left-sided acupuncture. The data of the normal group with right-sided acupuncture was also flipped. The number of flipped data was 13 out of 27 in the normal group, 10 out of 23 in the early group, 13 out of 19 in the later group, and 9 out of 22 in the recovered group. 

Group analysis and intergroup comparison were performed with the program of 3dttest++ with AFNI software. In order to reduce the influence of gender [34], age, acupuncture sensation [40], and other relevant factors as much as possible, in the group and intergroup analyses, we added the following variables as covariates including gender, age, severity of acupuncture sensation (soreness, numbness, heaviness, fullness, spreading, and aching), and whether or not the image was flipped. Monte Carlo simulation was done, and statistical significance was thresholded at *P* ≤ 0.01 (*z* = 2.575) and a minimum cluster size of 20 voxels with *α* ≤ 0.05% (3dClustSim, AFNI). Because that some obviously different activated areas were demonstrated as a single connected area in the original results, which mainly existed in the cerebellum, connected areas of different activations were manually segmented into different activations. The anatomical localization and labeling of the functional data were determined by both Talairach coordinates and direct inspections by more than three radiologists. 

## 3. Results

### 3.1. General Information

**Figure 2:**
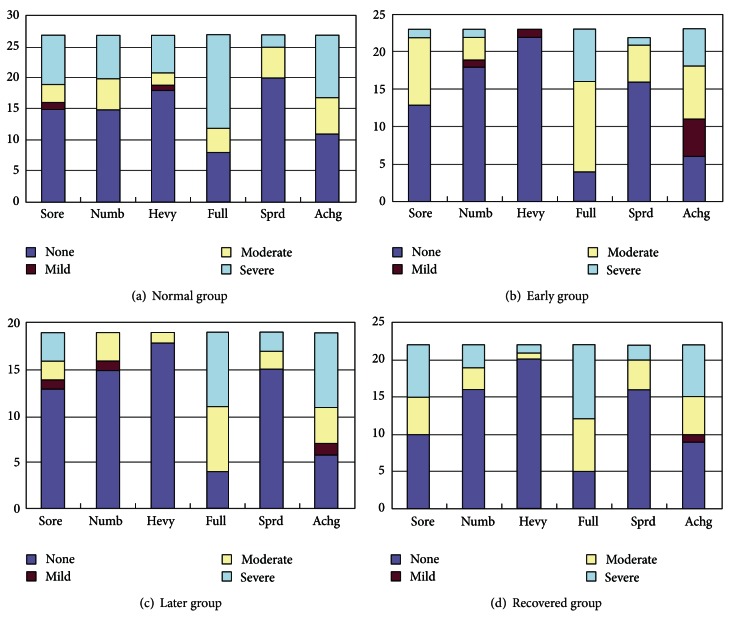
Demonstration of acupuncture sensation composition of different degrees in the four groups. The acupuncture sensations were labeled on the *x*-axis, including soreness (Sore), numbness (Numb), heaviness (Heav), fullness (Full), spreading (Sprd), and aching (Achg). The different degrees of sensations were marked with different colors as shown in the legend. The numbers on the *y*-axis indicated the cases for each kind of sensation.

Patients with Bell's palsy were classified into three groups according to the pathological stage, including the early group, later group, and recovered group. After grouping and exclusion, there were 23 cases in the early group (11 males, 12 females; mean age: 40.9 years, range 19–70 years), 19 cases in the later group (12 males, 7 females; mean age: 41.1 years, range 19–70 years), and 22 cases in the recovered group (12 males, 10 females; mean age: 37.9 years, range 19–63 years). Duration from onset was 7.0 ± 2.5 days in the early group, 34.6 ± 17.8 days in the later group, and 44.2 ± 25.6 days in the recovered group. The normal group was composed of 27 healthy volunteers (15 males, 12 females; mean age: 34.4 years, range 26–54 years). The HBS scale was as follows. In the early group, there were 4 cases of grade II, 10 cases of grade III, and 9 cases of grade IV. In the later group, there were 10 cases of grade II and 9 cases of grade III. The composition of acupuncture sensation in the four groups is demonstrated in Figure 2. 

### 3.2. Group Analysis Results of the Four Groups

**Figure 3:**
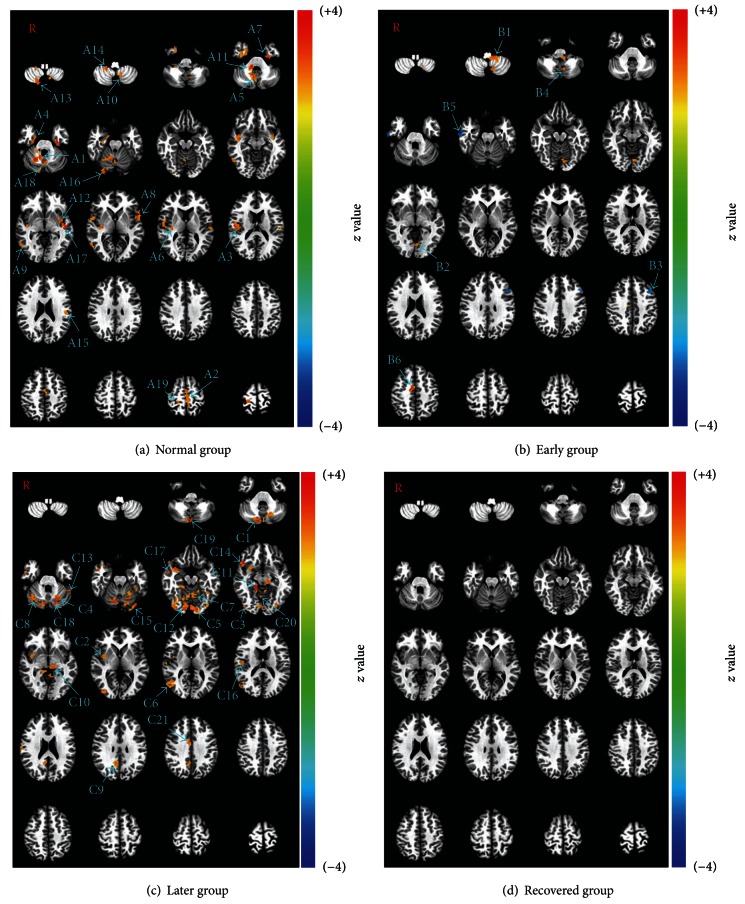
Demonstration of the group analysis results of the normal, early, later, and recovered groups (*P* ≤ 0.01, *α* ≤ 0.05, corrected with the Monte Carlo method). In the normal group, the activated brain areas spread over the whole brain, including lobule IX (A1), middle cingulate gyrus (A2), Rolandic operculum (A3), parahippocampal gyrus (A4), lobule IX (A5), insula (A6), parahippocampal gyrus (A7), inferior frontal gyrus (A8), inferior temporal gyrus (A9), lobule IX (A10), lobule III (A11), insula (A12), lobule VIIIa (A13), lobule VIIIb (A14), supramarginal gyrus (A15), lingual gyrus (A16), superior temporal gyrus (A17), lobule VIIa (A18), and postcentral gyrus (A19). In the early group, the activated areas became only a few, including lobule IX (B1 and B4), lingual gyrus (B2), and middle cingulate gyrus (B6), and there appeared deactivated areas in the middle frontal gyrus (B3) and inferior temporal gyrus (B5). In the later group, the activated areas became more obvious and more intense than those in the normal group which were shown in lobule VIIa (C1), superior temporal gyrus (C2), fusiform gyrus (C3), lobule VI (C4), lingual gyrus (C5), middle temporal gyrus (C6), culmen of cerebellum (C7), lobule VI (C8), posterior cingulate gyrus (C9), thalamus (C10), parahippocampal gyrus (C11), lingual gyrus (C12), fusiform gyrus (C13), inferior frontal gyrus (C14), fusiform gyrus (C15), insula (C16), middle temporal gyrus (C17), lobule VI (C18), vermis of the cerebellum (C19), lobule VI (C20), and middle cingulate gyrus (C21). In the recovered group, no activated areas were found above the threshold (*P* ≤ 0.01, *α* ≤ 0.05).

The group analysis demonstrated quite different activation patterns upon acupuncture among the four groups. In the normal group, extensive activations were shown in the areas of the SI, SII, insula, middle cingulate gyrus, parahippocampal gyrus, inferior frontal gyrus, inferior temporal gyrus, superior temporal gyrus, lingual gyrus, and extensive areas of the cerebellum including the vermis and lobules III, VII, VIII, and IX of the cerebellum (Table 1, Figure 3(a)). In the early group, far less activation was shown in the areas of the lingual gyrus, middle cingulate gyrus and lobule IX of the cerebellum, and there appeared deactivated areas in the middle frontal gyrus and inferior temporal gyrus (Table 2, Figure 3(b)). In the later group, the activated areas became more abundant, being mainly distributed in the lingual gyrus, fusiform gyrus, and cerebellum (Table 2, Figure 3(c)). In the recovered group, no activated area was found above the threshold (Monte Carlo correction, *P* ≤ 0.01, *α* ≤ 0.05% with a minimum cluster size of 20 voxels).

### 3.3. Intergroup Comparison between Patients and Normal Controls

**Figure 4:**
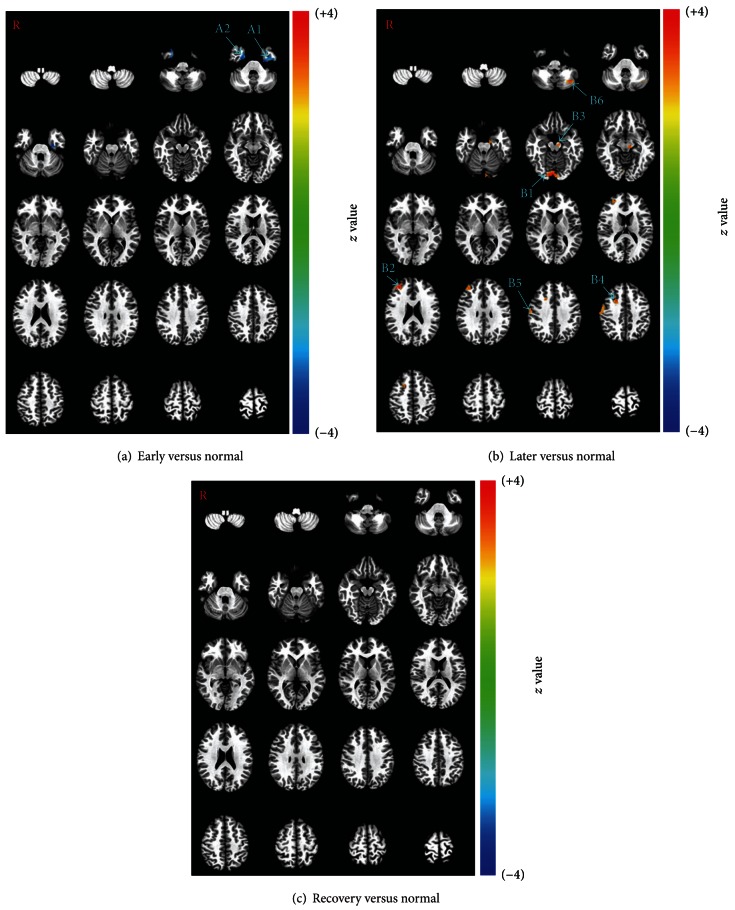
Intergroup comparison analysis results between the patients and the normal controls (*P* ≤ 0.01, *α* ≤ 0.05, corrected with the Monte Carlo method). With the comparison of early versus normal, decreased activations (early < normal) were demonstrated in the early group on both sides of parahippocampal gyrus (A1 and A2). With the comparison of later versus normal, increased activations (later > normal) were demonstrated in the later group in the lingual gyrus (B1), middle frontal gyrus (B2 and B4), thalamus (B3), precentral gyrus (B5), and lobule VIIa (B6). With the comparison of recovery versus normal, no activated areas were found above the threshold (*P* ≤ 0.01, *α* ≤ 0.05).

Although the group analysis results were extremely different between the normal group and the recovered group, the intergroup analysis did not demonstrate any significant difference between these two groups. However, there were significant differences between the unrecovered patients (early and later groups) and the normal controls (normal group). When compared with the normal group, the early group showed decreased activation on both sides of the parahippocampal gyrus (Table 3, Figure 4(a)). When compared with the normal group, the later group showed increased activation on both sides of the lingual gyrus, right premotor cortices, right primary motor area, and left thalamus (Table 3, Figure 4(b)). 

## 4. Discussion

This is the first report on the brain response to acupuncture at different stages of Bell's palsy, which would be helpful in exploring the brain responses to acupuncture more comprehensively. With this objective, we explored the differences in brain activations to acupuncture between normal controls and patients with Bell's palsy at different pathological stages. In this part of the discussion, we would first discuss the general tendency of the changes in the brain response to acupuncture and then focus on the following two questions, that is, what the altered areas were and what the changes might imply. 

### 4.1. Overall Tendency of the Changes in the Brain Response to Acupuncture

The group analysis of the four groups showed quite different results. The activated brain areas in the normal group spread over the whole brain, and there was no deactivated area (see Figure 3(a)), while the activated areas in the early group were only a few, and there also appeared two deactivated areas (see Figure 3(b)). The activated areas in the later group became more obvious and more intense than those of the normal group, which was especially true in the cerebellum and motor association cortex (see Figure 3(c)). In the recovered group, there were no activated or deactivated areas above the threshold with Monte Carlo correction (*P* ≤ 0.01, *α* ≤ 0.05%, and cluster size ≥ 20 voxels) (see Figure 3(d)). With the group analysis results, it was demonstrated that the brain response to acupuncture decreased in the early stage, increased again in the later stage, and decreased back in the recovered stage. 

The intergroup analysis results provided further evidence for the impression on the tendency of the changes in the activations. In comparison with the normal group, only decreased activations were demonstrated in the early group on both sides of the temporal cortex (see Figure 4(a)), while only increased activations were demonstrated in the later group (see Figure 4(b)). There were no significant changes in the activation between the normal group and recovered group although the group analysis results were quite different (see Figures 3(a) and 3(d)). Therefore, it could be seen that the overall tendency of the changes in the brain response to acupuncture was that activations were decreased in the early stages, increased in the later stages, and nearly returned to normal in the recovered group. The successive question should be what the specific changes in the brain response to acupuncture are and what the changes might imply.

### 4.2. Specific Changes in the Brain Response to Acupuncture

In order to uncover the acupuncture mechanism underlying the varied brain response to acupuncture in different pathological stages of Bell's palsy and normal controls, we should first have a look at the specific changes in the brain response to acupuncture at the different stages and try to find out why the brain activation was changed. 

#### 4.2.1. Visual Association Cortex

In the group comparison analysis of later versus normal, the activation of the lingual gyrus was demonstrated as being significantly increased in the later group (see Figure 4(b)). Moreover, the lingual gyrus was also one of the activated areas in the group analysis results of the early and later groups (see Figures 3(b) and 3(c)). Considering that patients with Bell's palsy at early and later stages always have some problems in closing their eyes and that the lingual gyrus plays an important role in visual perception, hence this brain area should be an important one in the pathological process of Bell's palsy as far as the brain response to acupuncture was concerned.

The lingual gyrus (BA18) is a dorsal part of the second visual cortex (V2) that is, the second major area in the visual cortex. Recent research has shown that V2 plays an important role in selective attention [59–64]. Increased activation of V2 in the later stage of Bell's palsy should be a sign of an increased role in selective attention of V2. The reason for the enhanced role in selective attention of V2 should result from incomplete eye closure which was the most important symptom in patients with Bell's palsy [56, 57]. According to the House-Brackmann facial nerve grading system, an HBS scale greater than or equal to grade IV represents incomplete eye closure, grade III is complete closure with effort, and grade II is complete closure with minimum effort. In other words, all of the subjects in the early and later groups had problems in closing their eyes to some degree. This should be the reason for changes in the activation of the lingual gyrus.

The changes in the visual association cortex, such as the parahippocampal gyrus, were demonstrated as decreased activations in the early group when comparing the early group with the normal one, which could be able to provide some supplemental evidence. The parahippocampal gyrus is a grey matter cortical region of the brain that surrounds the hippocampus. This region plays an important role in visual memory encoding and retrieval [65, 66]. Problems with eye closure in the early group could result in a continuous stimulus of light. As a compensatory reaction, the brain might have inhibited the role of the parahippocampal gyrus in visual memory. Therefore, it could be suggested that the problem of incomplete eye closure in patients with Bell's palsy could change the functional status of the visual association cortex, which in turn would affect the brain response to acupuncture.

#### 4.2.2. Motor Association Cortex

In the group comparison analysis of later versus normal, significantly increased activations in the later group were demonstrated (see Figure 4(b)) on the right middle frontal gyri (BA10 and BA8) and right precentral gyrus (BA4). The precentral gyrus (BA4) is the primary motor cortex, which works in association with other motor areas to plan and execute movements. There is a broadly somatotopic representation of the different body parts in the primary motor cortex in an arrangement called a motor homunculus. The increased activated area on BA4 in this study corresponded to the somatotopic representation of the face primary motor cortex (M1), which was supported by a previous study on Bell's palsy [55]. In this study on cortical reorganization in Bell's palsy, patients with Bell's palsy were instructed to move the left or right mouth angle up followed by a brief relaxation of the facial muscles to regain the starting position. The activated right M1 by this motor task in reference [55] was identical to the right precentral gyrus (BA4) activated by acupuncture stimulus in this study. The other two areas of increased activation were BA6 and BA10. BA6 is part of the dorsal premotor cortex, which plays an important role in the planning of complex, coordinated movements [67, 68], and BA10 is the most anterior lateral portion of the prefrontal cortex, which is activated by tasks that require integration of multiple relations [69]. 

All of the increased activations were located on the right side. Since that data collected from patients with left-sided facial palsy were mirror reversed across the midsagittal plane before the group analysis, all group analysis results were equivalent to the results of right-sided facial palsy. Therefore, it could be suggested that the changed brain responses to acupuncture were contralateral to the palsy area. These areas contralateral to the facial palsy have also been found as the areas of overactivity when patients with Bell's palsy were performing a facial movement task and the overactivity during the mouth movement was explained by the brain's compensatory reaction to the failure of facial muscle movement [55]. The key point is why the overactivity was likewise demonstrated during the task of the acupuncture stimulus. In fact, these areas have not been found to be activated by the acupuncture stimulus in healthy volunteers [70]. Therefore, the possible explanation is that the functional status of these motor-associated brain areas changed due to the facial palsy, and the altered brain functional status, in turn, affected the brain response to acupuncture.

#### 4.2.3. Cerebellum

Activation of the cerebellum was demonstrated in the group analysis of the normal, early and later groups (Figure 3), and it became extremely significant in the later group. In the group comparison analysis of later versus normal, increased activations in the left cerebellum were demonstrated in the later group (see Figure 4(b)). The cerebellum is a region of the brain that plays an important role in motor control. It may also be involved in some cognitive functions such as attention and language, and in regulating fear and pleasure responses, but its movement-related functions are the most solidly established [71–81]. The cerebellum does not initiate movement, but it contributes to coordination, precision, and accurate timing. It receives input from sensory systems of the spinal cord and from other parts of the brain and integrates these inputs to fine tune motor activity [78]. A study of Bell's palsy with fMRI also suggested that the cerebellum played an important role in the long-term adaptation to transient pathological sensorimotor processing [82]. In other words, as a compensatory reaction to facial muscle movement failure, the functional status of the cerebellum was changed in the pathological process of Bell's palsy and the changed cerebellar response to acupuncture should be due to the changed functional status of the cerebellum.

### 4.3. Underlying Mechanism of Changing the Brain Response to Acupuncture

As demonstrated in the group analysis results (see Table 1 and Figure 3(a)), the normal group showed extensive activation in the brain, which not only included the somatosensory association cortex, such as the SI, SII, insula, and middle cingulate cortex, but also included the visual association cortex, motor association cortex and extensive cerebellar areas. Practically, acupuncture might be a kind of specialized and complicated stimulus of sensation. The spinothalamic system has been viewed as the major pathway for transmitting stimulus information to the cerebral cortex. The precise location and relative strength of the input to the cortical spinothalamic targets could be defined only recently using transsynaptic viral transport from spinothalamic neurons in the cord [83]. While spinothalamic projections to the primary somatosensory cortex appeared to receive less than 5% of the spinothalamic system input, the vast majority of spinothalamic cortical targets were found in the posterior insular cortex (granular insula, *∼*40%), the medial parietal operculum (*∼*30%), and the motor sections of the midcingulate cortex (*∼*24%). These findings were consistent with the brain responses to acupuncture in many acupuncture fMRI studies where the SII, insula, and middle cingulate cortex were the most frequently activated areas [13, 15, 42, 70, 84]. In fact, the activation of these areas was also frequently reported in fMRI studies of ordinary nerve stimulation [85, 86]. Therefore, we could deduce that acupuncture is basically a kind of cutaneous sensation stimulus. This is so because the human brain is a very complicated network in which various regions are closely and functionally connected [87], so the stimulus of acupuncture could not only activate the somatosensory cortex, but it could also activate many other regions functionally connected to the somatosensory cortex, such as the vision association areas, motor association cortex and cerebellum. Varied brain responses to acupuncture demonstrated in this paper provided a good example for this assumption.

As stated above, we demonstrated that the brain response to acupuncture was varied at different stages of Bell's palsy, and most of the changed activation could be explained as the brain compensatory reaction to a relevant stimulus, such as an unpleasant visual stimulus, or a functional failure such as facial muscle paralysis. As a matter of fact, all of these compensatory reactions of the brain were substantially demonstrated as the changes in the brain functional status. Therefore, we deduced that the changes in different stages of Bell's palsy might, at least in part, be the results of an altered brain functional status. In other words, because the functional status of these brain areas was weakened or enforced, the brain responses to acupuncture were demonstrated as decreased or increased activations. On the other hand, the fact that the brain response to acupuncture was different at different pathological statuses, to some degree, is in accordance with the traditional Chinese medical theory that acupuncture plays a homeostatic role [88] and thus may have a different effect on the patient populations with a pathological imbalance, as compared to healthy individuals. However, whether or not the different effects are beneficial could not be demonstrated in this paper and should be investigated further.

Based on the deduction stated above, we therefore proposed that the brain response to acupuncture was probably dependent on the brain functional status. This proposition might not be consistent with some other viewpoints related to acupuncture's underlying mechanism. However, if this proposition could be proven, a lot of discrepancy could be resolved, such as individual variability and varied brain response to acupuncture in different diseases. For example, there were no deactivations demonstrated in healthy control in this study, but some other studies demonstrated extensive deactivations in the limbic-paralimbic neocortical network in healthy volunteers [32, 39, 43]. The reasons for this inconsistency could be very complicated, which could be related to the stimulus paradigm, control design, acupuncture manipulation, and methodological issues. However, the difference in recruited subjects and their different brain functional statuses might be one of the reasons that should be taken into account. 

Although the proposition seemed reasonable, it had not been convincingly proven in the paper. In order to further provide evidence for the proposition, some further investigations should be done, such as functional connectivity analysis in patients with Bell's palsy to find whether or not the functional connectivity had really been weakened or enforced in the related brain areas. More importantly, in order to establish the specific benefit of acupuncture, a control group with a natural course of the disease (without treatment) or a control group with conventional corticosteroid treatment would be necessary. At the same time, a better study design and statistical methods should be adopted in further investigations to overcome the limitations of the current study.

### 4.4. Limitations of the Present Study

There were some limitations in this study. Firstly, because of the unequal compliance from the patient, this study was not strictly a longitudinal study with repeated measures design. The patients in the early, later, and recovered groups were partly overlapped, but not strictly paired. Considering that independent data is necessary for ANOVA and a *t*-test analysis, we simply compared different stages of the patients with healthy controls, but not between different stages of the patients, which might potentially influence the reliability of our results and should be clarified in future studies. Secondly, by the same strategy that some previous studies had used [44, 55], the data collected from patients with left-sided facial palsy were flipped across the midsagittal plane before group and intergroup analyses in the current study, and we added the factor of whether or not the data were flipped into the covariates when performing the group analysis. Nevertheless, it was still of bias when we took the factor of laterality into account. To overcome this limitation, more patients should be recruited in a further study. Thirdly, because there was no sham acupuncture control included in this study, the conclusions from this study might not be necessarily specific to acupuncture; for example, the differences in the brain response to acupuncture may be similarly observed in response to other somatic stimulation, such as sham acupuncture. It would be better to include a control task of sham acupuncture in the further investigations. Fourthly, the paradigms used in this study are not representative of typical clinical practice, where more than one acupuncture point is penetrated for longer periods and repeated 3 times per week for several weeks. Moreover, some subjects were not acupuncture naïve, and the number of acupuncture treatments received in the later and recovered groups was different across subjects. Therefore, the changed brain response to acupuncture might not definitely result from the pathological status. The acupuncture cumulative effect or the psychological effect of the acupuncture stimulus could not be fully excluded in this study. Therefore, more strict control of relevant factors should be necessary in the future study.

## 5. Conclusions

We have presented evidence that acupuncture activations in the brain of patients with Bell's palsy differed from those in healthy controls, and patients with Bell's palsy at different pathological stages had different brain responses to the acupuncture stimulus. The brain response to acupuncture was decreased in the early stages, increased in the later stages, and nearly returned to normal in the recovered group. The changed activation areas included the visual association cortex, motor association cortex, and cerebellum. All of these changes in the brain response to acupuncture might be relevant to the differences in the brain functional status. Therefore, we proposed that the brain response to acupuncture at different pathological stages in patients with Bell's palsy is probably dependent on the brain functional status, while further evidence was still needed to support our proposition.

## Figures and Tables

**Table 1:** Group analysis results of brain activation areas in the normal group.

Group analysis	Regions (BA)	Side	Peak *z* value	Coordinate (Talairach)			Voxel size
				Peak *x*	Peak *y*	Peak *z*	
	Lobule IX of cerebellum	R	4.11	−10.5	49.5	−27.5	92
	Middle cingulate gyrus (6)	L	3.10	7.5	28.5	59.5	78
	Rolandic operculum (43), SII	R	3.51	−49.5	16.5	17.5	71
	Parahippocampal gyrus (38)	R	3.57	−22.5	−7.5	−33.5	69
	Lobule IX of cerebellum	R	3.51	−7.5	55.5	−30.5	58
	Insula (13)	R	3.59	−31.5	19.5	8.5	57
	Parahippocampal gyrus (20)	L	4.05	28.5	13.5	−27.5	43
	Inferior frontal gyrus (45)	L	3.93	55.5	−16.5	5.5	38
	Inferior temporal gyrus (37)	R	3.60	−55.5	55.5	−3.5	37
Normal group	Lobule IX of cerebellum	L	3.05	13.5	49.5	−42.5	31
	Lobule III of cerebellum	R	4.04	−10.5	34.5	−30.5	29
	Insula (13)	L	3.35	34.5	4.5	−3.5	28
	Lobule VIIIa of cerebellum	R	3.30	−13.5	70.5	−48.5	27
	Lobule VIIIb of cerebellum	R	3.52	−19.5	37.5	−45.5	26
	Supramarginal gyrus (13), SII	L	2.97	49.5	22.5	23.5	25
	Lingual gyrus (18)	R	3.42	−25.5	82.5	−18.5	23
	Superior temporal gyrus (22)	L	3.63	46.5	16.5	−0.5	22
	Lobule VIIa of cerebellum	R	3.62	−16.5	79.5	−21.5	21
	Postcentral gyrus (3), SI	R	3.20	−19.5	34.5	62.5	20

BA: Brodmann area; L: left; R: right; SI: primary somatosensory cortex; SII: secondary somatosensory cortex; the threshold was set to *P* ≤ 0.01, *α* ≤ 0.05 (corrected with the Monte Carlo method).

**Table 2:** Group analysis results of brain activation areas in each of the patient groups.

Group analysis	Regions (BA)	Side	Peak *z* value	Coordinate (Talairach)			Voxel size
				Peak *x*	Peak *y*	Peak *z*	
	Lobule IX of the cerebellum	L	3.51	7.5	37.5	−39.5	49
	Lingual gyrus (18)	L	3.32	7.5	73.5	−9.5	35
Early group	Middle frontal gyrus (8)	L	−3.45	37.5	−16.5	38.5	34
	Lobule IX of the cerebellum	L	3.45	7.5	58.5	−39.5	30
	Inferior temporal gyrus (20)	R	−3.54	−61.5	13.5	−18.5	28
	Middle cingulate gyrus(24)	R	3.85	−7.5	7.5	47.5	28

	Lobule VIIa of the cerebellum	R	3.66	−16.5	79.5	−21.5	98
	Superior temporal gyrus (38)	R	3.39	−34.5	−4.5	−12.5	85
	Fusiform gyrus (19)	R	3.24	−28.5	64.5	−15.5	71
	Lobule VI of the cerebellum	L	3.48	22.5	61.5	−24.5	68
	Lingual gyrus (18)	L	3.49	7.5	85.5	−15.5	59
	Middle temporal gyrus (19)	R	3.16	−43.5	61.5	14.5	58
	Culmen of the cerebellum	L	3.26	4.5	43.5	−3.5	54
	Lobule VI of the cerebellum	R	3.79	−28.5	64.5	−21.5	50
	Posterior cingulate gyrus (31)	R	3.13	−7.5	49.5	29.5	50
	Thalamus	L	3.61	10.5	25.5	−3.5	49
Later group	Parahippocampal gyrus (30)	R	4.78	−13.5	37.5	−6.5	49
	Lingual gyrus (18)	R	3.35	−13.5	82.5	−12.5	37
	Fusiform gyrus (36)	L	3.80	49.5	46.5	−24.5	36
	Inferior frontal gyrus (47)	R	4.17	−43.5	−19.5	-6.5	35
	Fusiform gyrus (19)	L	4.48	37.5	76.5	−18.5	32
	Insula (13)	R	3.07	−43.5	19.5	17.5	29
	Middle temporal gyrus (21)	R	3.92	−49.5	1.5	−15.5	28
	Lobule VI of the cerebellum	L	3.54	16.5	70.5	−21.5	27
	Vermis of the cerebellum	L	4.03	−1.5	76.5	−33.5	25
	Lobule VI of the cerebellum	R	2.95	−13.5	58.5	−15.5	22
	Middle cingulate gyrus (24)	R	3.18	−4.5	4.5	35.5	20

BA: Brodmann area; L: left; R: right; SII: secondary somatosensory cortex. The threshold was set to *P* ≤ 0.01, *α* ≤ 0.05 (corrected with the Monte Carlo method). No activated area was found above the threshold in the recovered group.

**Table 3:** Intergroup comparison analysis results of brain activation differences between patients at three different pathological stages with normal controls (early versus normal, later versus normal, and recovery versus normal).

Inter group	Regions (BA)	Side	Peak *z* value	Coordinate (Talairach)			Voxel size
				Peak *x*	Peak *y*	Peak *z*	
Early versus normal	Parahippocampal gyrus (28)	L	−3.81	25.5	13.5	−27.5	45
	Parahippocampal gyrus (36)	R	−3.57	−25.5	7.5	−33.5	33

	Lingual gyrus (18)	R	3.73	−4.5	82.5	−12.5	69
	Middle frontal gyrus (10)	R	4.08	−31.5	−40.5	23.5	46
Later versus normal	Thalamus	L	3.19	10.5	19.5	−6.5	33
	Middle frontal gyrus (6)	R	3.62	−19.5	−13.5	38.5	28
	Precentral gyrus (4)	R	3.27	−55.5	13.5	38.5	28
	Lobule VIIa of the cerebellum	L	3.62	40.5	61.5	−33.5	22

BA: Brodmann area; L: left; R: right. The threshold was set to *P* ≤ 0.01, *α* ≤ 0.05 (corrected with the Monte Carlo method). No significant differences were found between the normal group and the recovered group.
